# Polytropic Influence of *TRIB3* rs2295490 Genetic Polymorphism on Response to Antihypertensive Agents in Patients With Essential Hypertension

**DOI:** 10.3389/fphar.2019.00236

**Published:** 2019-03-27

**Authors:** Jiecan Zhou, Fazhong He, Bao Sun, Rong Liu, Yongchao Gao, Huan Ren, Yan Shu, Xiaoping Chen, Zhaoqian Liu, Honghao Zhou, Sheng Deng, Heng Xu, Jianmin Li, Linyong Xu, Wei Zhang

**Affiliations:** ^1^Department of Clinical Pharmacology, Xiangya Hospital, Central South University, Changsha, China; ^2^Pharmacogenetics Research Institute, Institute of Clinical Pharmacology, Hunan Key Laboratory of Pharmacogenetics, Central South University, Changsha, China; ^3^National Clinical Research Center for Geriatrics, Xiangya Hospital, Central South University, Changsha, China; ^4^Pharmacy Department, The First Affiliated Hospital, University of South China, Hengyang, China; ^5^Department of Pharmaceutical Sciences, School of Pharmacy, University of Maryland, Baltimore, MD, United States; ^6^Department of Pharmacy, Xiangya Hospital, Central South University, Changsha, China; ^7^Department of Laboratory Medicine, Precision Medicine Center, and Precision Medicine Key Laboratory of Sichuan Province, Collaborative Innovation Center, West China Hospital, Sichuan University, Chengdu, China; ^8^Department of Respiratory Medicine, Hunan Provincial People’s Hospital, The First Affiliated Hospital of Hunan Normal University, Changsha, China; ^9^Department of Epidemiology and Health Statistics, Xiangya School of Public Health, Central South University, Changsha, China

**Keywords:** *TRIB3*, rs2295490, polymorphism, antihypertensive agents, essential hypertension

## Abstract

Tribbles homolog 3 (*TRIB3*) mediating signaling pathways are closely related to blood pressure regulation. Our previous findings suggested a greater benefit on vascular outcomes in patients carrying *TRIB3* (251, A > G, rs2295490) G allele with good glucose and blood pressure control. And *TRIB3* (rs2295490) AG/GG genotypes were found to reduce primary vascular events in type 2 diabetic patients who received intensive glucose treatment as compared to those receiving standard glucose treatment. However, the effect of *TRIB3* genetic variation on antihypertensives was not clear in essential hypertension patients. A total of 368 patients treated with conventional dosage of antihypertensives (6 groups, grouped by atenolol/bisoprolol, celiprolol, doxazosin, azelnidipine/nitrendipine, imidapril, and candesartan/irbesartan) were enrolled in our study. Genetic variations were successfully identified by sanger sequencing. A linear mixed model analysis was performed to evaluate blood pressures among *TRIB3* (251, A > G) genotypes and adjusted for baseline age, gender, body mass index, systolic blood pressure (SBP), diastolic blood pressure (DBP), total cholesterol and other biochemical factors appropriately. Our data suggested that *TRIB3* (251, A > G) AA genotype carriers showed better antihypertensive effect than the AG/GG genotype carriers [*P* = 0.014 for DBP and *P* = 0.042 for mean arterial pressure (MAP)], with a maximal reduction of DBP by 4.2 mmHg and MAP by 3.56 mmHg after azelnidipine or nitrendipine treatment at the 4th week. Similar tendency of DBP-change and MAP-change was found for imidapril (ACEI) treatment, in which marginally significances were achieved (*P* = 0.073 and 0.075, respectively). Against that, we found that *TRIB3* (251, A > G) AG/GG genotype carriers benefited from antihypertensive therapy of ARBs with a larger DBP-change during the period of observation (*P* = 0.036). Additionally, stratified analysis revealed an obvious difference of the maximal blood pressure change (13 mmHg for the MAP between male and female patients with AA genotype who took ARBs). Although no significant difference in antihypertensive effect between *TRIB3* (251, A > G) genotypes in patients treated with α, β-ADRs was observed, we found significant difference in age-, sex-dependent manner related to α, β-ADRs. In conclusion, our data supported that *TRIB3* (251, A > G) genetic polymorphism may serve as a useful biomarker in the treatment of hypertension.

## Introduction

Essential hypertension (EH) can be defined as a rise in blood pressure for unknown cause. It can increase risks for cerebral, cardiac, and renal events. It usually clusters with other cardiovascular risk factors such as aging, being overweight, insulin resistance, diabetes, and hyperlipidaemia (Messerli et al., 2007; Sun et al., 2019). Human tribbles homolog 3 (*TRIB3*) is organized as four exons, encoding a 358-amino-acid protein. TRIB3 proteins disrupts insulin signaling by binding directly to Akt and blocking activation of the kinase, which contributes to insulin resistance and other physiological processes of vessel (Du et al., 2003; Prudente and Trischitta, 2015). AKT mediates the phosphatidylinositol 3′-kinase-protein kinase B-endothelial nitric oxide (NO) synthase (PI3K-AKT-eNOS)-dependent pathway in the cardiovascular protective effect of insulin, which is crucial for NO synthase activation, eventually leading to increased NO production, vasodilation and blood flow (Yu et al., 2011). Other signaling molecules such as bone morphogenetic proteins type II receptor (BMRPII) and bone morphogenetic proteins (BMP) also interact with TRIB3 and modulate the vascular effect (Kiss-Toth et al., 2004; West et al., 2004; Chan et al., 2007). Accumulating evidences supported that some drugs or active materials affect vascular functions by regulating *TRIB3*. For example, the amiloride derivative phenamil promotes contractile phenotype of vascular smooth muscle cells (vSMCs) by activating *TRIB3* gene transcription (Chan et al., 2011; Kim et al., 2014). Homocysteine, which is significantly associated with hypertension could up-regulate the expression of *TRIB3*, thus leading to the endothelial dysfunction (Chan et al., 2011; Zou et al., 2011).

A single nucleotide polymorphism (SNP) variant in human *TRIB3* exon 2, which results in a glutamine (Q) to arginine (R) missense mutation in a conserved motif at position 84, confers stronger Akt binding, resulting in reduced Akt phosphorylation (Fischer et al., 2017). Since Prudente S first reported the relationship between *TRIB3* missense Q84R (rs2295490) polymorphism and insulin resistance or its related cardiovascular events (Prudente et al., 2005), numerous clinical studies have confirmed the risk of genetic variant associated with vascular events, especially the deleterious role of *TRIB3* R84 (Gong et al., 2009; Formoso et al., 2011; Prudente et al., 2015; Zhang et al., 2015). Among patients with metabolic syndrome and the control group, patients with *TRIB3* RR84 genotype had significantly higher level of serum semaphorin 3E than those with QQ84 or QR84 genotypes (Qin et al., 2017), individuals with RR84 genotype also showed further decreased serum obestatin. Increased serum semaphorin 3E or decreased serum obestatin might in part exacerbate insulin resistance and carotid atherosclerosis (Cui et al., 2012). In human endothelial cells, data demonstrate that the *TRIB3* R84 variant impairs insulin signaling and NO production (Andreozzi et al., 2008). These vascular disease-related studies suggested a possible link between *TRIB3* and EH.

Studies showed that imidapril mediated various actions, such as vasodilation, anticoagulation, hypotension, atherosclerosis and other cardiovascular protection via bradykinin-eNOS-NO pathway (Kobayashi et al., 2000; Chen et al., 2003). Celiprolol triggered PI3K-Akt and increased eNOS expression (Kobayashi et al., 2003), and doxazosin also increased the expression of *eNOS* accompanying with NO production via cAMP/cGMP/adrenalin signaling pathway (Stojkov et al., 2013, 2014). Azelnidipine significantly increased *eNOS* expression levels in the brain as well as in the heart and aorta (Kimura et al., 2007). Zhang, et al. demonstrated that valsartan significantly down-regulated the expression of *TRIB3* mRNA level and improved the cardiac function in rats with diabetic cardiomyopathy (Zhang et al., 2006).

These data indicate that *TRIB3* has an integral role in regulating the blood pressure, and its genetic variation (251, A > G) may cause a variability of antihypertensive drug therapy. Our previous findings have suggested a greater benefit on vascular outcomes in patients carrying *TRIB3* (rs2295490) G allele with good glucose and blood pressure control (He et al., 2016). *TRIB3* (rs2295490) AG/GG genotypes were found to reduce primary vascular events in patients who received intensive glucose treatment as compared to those receiving standard glucose treatment in type 2 diabetic patients (He et al., 2018). However, to our knowledge, there is no study to evaluate the association between *TRIB3* Q84R polymorphism and the effect of antihypertensive drugs in patients with EH.

## Patients and Methods

### Patients

**Figure 1:**
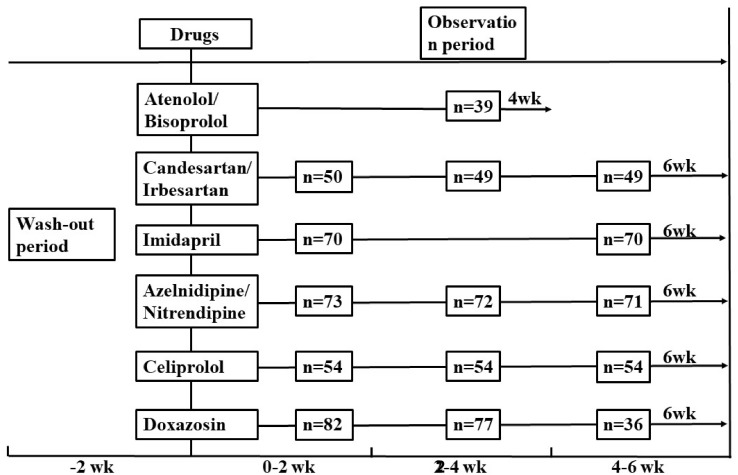
Study protocol and visit time to 368 patients with related drugs in the study. wk, week.

The study protocol was approved by the Ethical Committee of the Institute of Clinical Pharmacology, Central South University (Hunan, China). Clinical study was registered at the Chinese Clinical Trial Register with the registration number ChiCTR-RO-12002612^1^. All the patients participating in the study were given written informed consent. The clinical data and DNA samples were graciously provided by Shanghai Institute of Hypertension, Ruijin Hospital affiliated with Shanghai Jiao Tong University. According to study design, all the patients fulfilled the following inclusion and exclusion criteria: male or female patients, heart rates within 55∼90 beats/min, systolic blood pressure (SBP) ≥ 140 mmHg and/or diastolic blood pressure (DBP) ≥ 90 mmHg were included. Patients with secondary hypertension, coronary heart disease, diabetes, obesity (BMI.30 kg/m^2^), stroke, renal or liver dysfunction, malignant tumor or pregnancy and those whose blood pressure measurements above 180/110 mmHg or remaining lower than 140/90 mmHg during the wash-out period, were withdrawn from the study. In our study, a total of 368 unrelated subjects with primary mild to moderate EH, aged from 26 to 81 years were recruited for genetic screening. All the patients were enrolled after a run-in period of 2 weeks and assigned to receive the drugs for 4 weeks or more. The patients were followed up every 2 weeks at the weekend, and details of the protocol were shown in Table 1. According to the study protocol, 86 samples were excluded from 454 eligible patients due to the small sample size, not contactable and fail to meet inclusion criteria. At last, a total of 368 patients were included in our study. The medications include β-adrenoceptor antagonists (β-ADRs, atenolol, bisoprolol and celiprolol), α-adrenoceptor antagonist (α-ADR, doxazosin), calcium channel blockers (CCBs, azelnidipine and compound nitrendipine), ACE-inhibitor (ACEI, imidapril) and angiotensin receptor blocker (ARB, candesartan and irbesartan). All patients received monotherapy. Details are shown in Figure 1.

**Table 1:** The protocols of the related drugs to a total of 368 eligible patients in the study.

	Sample	Treatment	Case
Drugs and dose	size (N)	course (W)	report
Celiprolol (200 mg/d)	54	6	ETW
Atenolol (25 mmg/d)/ bisoprolol (5 mmg/d)	39	4	EQW
Doxazosin (2 mmg/d)	82	6	ETW
Azelnidipine (2 mmg/d)/nitrendipine + atenolol (5 + 10 mmg/d)	73	6	ETW
Candesartan (8 mmg/d) + irbesartan (150 mmg/d)	50	6	ETW
Imidapril (5 mmg/d)	70	6 or 8	ETW or EQW

*N = number; W = week; ETW = every two weeks; EQW = every four weeks*.

### Blood Pressure and Heart Rate Measurement

Blood pressure determinations were performed on the morning after a light breakfast with subjects in the seated position and following a 30 min quiet resting period. Blood pressure and heart rate were measured by trained nurses, with an automatic blood pressure monitor with intellisense, which allows the detection of alteration of the heart rate by greater than or equal to beat/min and the blood pressure by greater than or equal to 1 mmHg. Detected results were then validated by supervisors with the mercury sphygmomanometer. The blood pressure values were determined by the average of three measurements taken every 10 min. Values of SBP and DBP were defined by Korotkoff phase I and V, respectively. Pulse pressure was calculated as PP = (SBP- DBP); mean arterial pressure (MAP) was calculated as MBP = DBP + (PP/3) (Wain et al., 2011).

### Genotyping Procedure for *TRIB3*

Tribbles homolog 3 (251, A > G) genotypes were determined by sanger sequencing. The PCR primers for *TRIB3* (251, A > G) were shown as follows: the sense primer, 5′GTTGCCCCTGAGCCCACCTACT3′; and the antisense primer, 5′TCCCTGGATGCTTCCCCACTAA3′. The product length was 286 bp. The reaction mixture (25 μl) contained: 10 × PCR buffer (2.5 μl), 10 × dNTP (2.5 μl), 10 μM for each of the sense and antisense primers (0.5 μl), H_2_O (16.8 μl), gDNA (2 μl), Taq polymerase (0.2 μl). Temperature cycling was proceeded as follows: initial denaturation for 5 min at 94°C, followed by 36 cycles of denaturation 30 s at 94°C, annealing at 57°C for 30 s, and elongation at 72°C for 30 s, and a terminal extension for 5 min. Genotypes were determined without knowledge of the status of patients, Ten percent blinded, random DNA samples from the patients were genotyped twice, with 100% concordance. And sequencing was assisted by Shanghai Majorbio Bio-pharm Technology Company.

### Statistical Analysis

All the analyses were performed with SPSS (version 19.0 for windows; Chicago, IL, United States). Allele frequencies were determined by the genotypes of all the participants. Continuous data were presented as mean values ± standard error (SE), and categorical variable was shown as frequencies and percentages. The Hardy–Weinberg equilibrium analysis was carried out for the genotypes of participants using the χ^2^-test. Difference in baseline characteristics among the phenotypes were assessed by independent-samples *T*-test.

The linear mixed model was used to examine the efficacy of *TRIB3* (251, A > G). This allowed the use of time-dependent and time-independent covariates within the model to be analyzed. Furthermore, this model has been found to work well for small sample sizes with missing data (Patel et al., 2007; Chen et al., 2015). The outcome measures included ΔSBP, ΔDBP and ΔMAP. These measures were analyzed individually, with the main effects of genotype, follow-up time and their interaction, as well as subjects’ intercept serving as random effects. The best fit model was decided after checking the smallest Akaike Information Criterion (AIC), and the Schwarz Bayesian Criterion (BIC) values. The covariance structure adopted for the final model was the Unstructured (UN) which means each variance and each covariance is estimated uniquely from the data. Models were additionally adjusted for the fixed effects of baseline age, sex, body mass index (BMI), SBP, DBP, total cholesterol, heart rate, triglyceride, high-density lipoprotein (HDL), low-density lipoprotein (LDL), fasting blood-glucose (FBG) and other biochemical factors appropriately. The linear mixed model analysis was done by using the PASW 19.0 for Windows (SPSS Inc., Chicago, IL, United States). Functions in nlme package of R statistical computing environment (R Foundation —for Statistical Computing, Vienna, Austria) were used to confirm the analysis. Stratified Analysis was used to address whether significant interaction was present between age-genotype and sex-genotype. A two-tailed *P*-value < 0.05 was considered significant.

## Results

### Baseline Characteristics, Genotyping Results and Outline of Major Findings

In this study, 368 patients were genotyped unambiguously for *TRIB3* (251, A > G) genetic variation. Our data showed that the frequency of *TRIB3* (251, A > G) G allele was 18.5% in Chinese population. Genotype frequencies of *TRIB3* (251, A > G) AA, AG and GG were 66.0, 31.0 and 3.0%, respectively. All the allele frequencies were in Hardy-Weinberg equilibrium (*P* > 0.05). Owing to the low frequency of the GG genotype, we combined the AG and GG genotypes as one group for the following analysis. Baseline characteristics of the patients stratified by *TRIB3* genotypes are shown in Table 2. No significant differences between AA and AG+GG genotype groups in age, BMI, HR, BP or other clinical characteristics, such as FBG, triglyceride, cholesterol, HDL and LDL, etc. were observed.

**Table 2:** Baseline characteristics of patients with essential hypertension stratified by *TRIB3* genotype.

Variable	*TRIB3*		*P*-value
	AA (n)	AG+GG (n)	
Age, year	57.0 ± 8.4(243)	56.5 ± 8.6(125)	0.524
BMI, kg/m^2^	25.2 ± 3.0(242)	25.1 ± 3.6(125)	0.723
HR, bpm	75.2 ± 7.5(243)	75.1 ± 7.3(125)	0.781
SBP, mm Hg	149.3 ± 10.3(243)	151.2 ± 10.5(125)	0.485
DBP, mm Hg	98.0 ± 4.3(243)	98.3 ± 4.8(125)	0.485
MAP, mm Hg	115.1 ± 5.3(243)	115.9 ± 5.6(125)	0.388
PP, mm Hg	51.3 ± 9.3(243)	52.8 ± 9.7(125)	0.100
ALT, umol/L	31.5 ± 17.3(212)	31.8 ± 27.7(101)	0.336
BUN, mmol/L	5.2 ± 1.2(222)	6.5 ± 9.3(109)	0.471
UCr, mmol/L	79.8 ± 16.6(237)	79.1 ± 17.3(122)	0.610
UA, mmol/L	320.4 ± 80.5(180)	328.1 ± 89.6(82)	0.246
FBG, mmol/L	5.2 ± 0.98(210)	5.4 ± 1.2(105)	0.739
TG, mmol/	1.78 ± 1.6(236)	2.33 ± 1.3(120)	0.653
CHO, mmol/L	5.1 ± 1.0(237)	5.4 ± 1.3(121)	0.101
HDL, μmol/L	1.4 ± 0.3(221)	1.4 ± 0.3(111)	0.740
LDL, umol/L	3.4 ± 1.1(144)	3.6 ± 1.0(78)	0.374

*Data expressed as mean ± SD. BMI indicates body mass index; HR, heart rate; SBP, systolic blood pressure; DBP, diastolic blood pressure; PP, pulse pressure; MAP, mean arterial pressure; ALT, alanine aminotransferase; BUN, blood urea nitrogen; UCr, urine creatinine; UA, uric acid; FBG, fasting blood-glucose; TG, triglyceride; CHO, cholesterol; HDL, high-density lipoprotein; LDL, low density lipoprotein*.

### Different Pressure Response to *TRIB3* (251, A > G) for Antihypertensive Drugs

**Figure 2:**
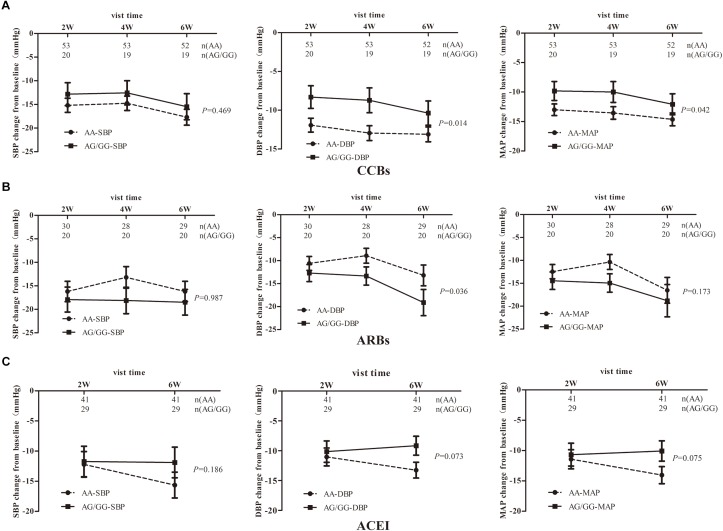
The relationship between *TRIB3* (251, A > G) genetic polymorphism and the hypotensive effects of the antihypertensives monotherapy in EH patients. Panels **(A–C)** respectively depicts that BP-changes from baseline in EH patients carrying the *TRIB3* (251, A > G) AA and AG/GG genotypes after treatment with the drugs of CCBs, ARBs and ACEIs for 6 weeks. *P*-value were calculated by the linear mixed model and adjusted for baseline age, BMI, gender, SBP, DBP, total cholesterol, heart rate, triglyceride, HDL, LDL, FBG and other biochemical factors appropriately. Error bar indicated 95% confidence interval.

**Figure 3:**
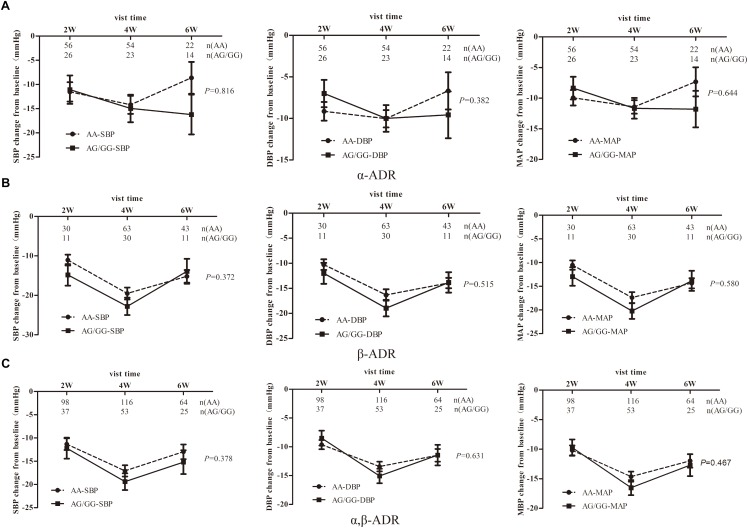
The relationship between *TRIB3* (251, A > G) genetic polymorphism and the hypotensive effects of α,β-ADRs monotherapy in EH patients. Panels **(A–C)** respectively depicts that BP- changes from baseline in EH patients carrying the *TRIB3* (251, A > G) AA and AG/GG genotypes after treatment with α-ADR, β-ADR and α, β-ADRs for 6 weeks. *P*-value were adjusted for baseline age, BMI, gender, SBP, DBP, total cholesterol, heart rate, triglyceride, HDL, LDL, FBG and other biochemical factors appropriately. Error bar indicated 95% confidence interval.

As shown in Figure 2A, the DBP-change and MAP-change from baseline response to *TRIB3* (251, A > G) variant was significantly reduced (AA genotype compared to AG/GG genotype carriers) after CCBs administration. A similar tendency of DBP-change and MAP-change was found for imidapril (ACEI) treatment (Figure 2C), in which a marginally significant difference was achieved (*P* = 0.073 and 0.075, respectively). Inversely, DBP change from baseline after ARBs prescription was significantly increased for the AA genotype carriers compared to AG/GG genotype (Figure 2B). In the stratified analysis, there were significant differences in BP-change (blood pressure, include SBP or DBP or MAP) of AG/GG carriers between older and younger age or male and female (Supplementary Figures S1, S2). Nevertheless, no association between *TRIB3* (251, A > G) genetic polymorphism and α-ADR or β-ADR drug response was observed in our study cohort (Figure 3A,B).

### Age-, Sex-Dependent Manner Related to α, β-ADRs on Blood Response

**Figure 4:**
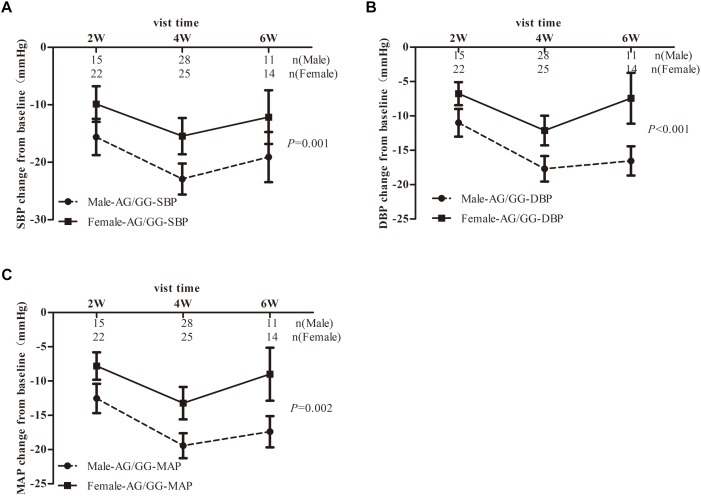
Blood pressure response to α,β-ADR blocker therapy in EH patients stratified by sex dependent of *TRIB3* genotype. Panels **(A–C)** respectively depicts that SBP-, DBP-, MAP- changes from baseline in EH patients carrying the *TRIB3* (251, A > G) AA and AG/GG genotypes after treatment with α, β-ADRs for 6 weeks. *P*-value were adjusted for baseline age, BMI, SBP, DBP, total cholesterol, heart rate, triglyceride, HDL, LDL, FBG and other biochemical factors appropriately. Error bar indicated 95% confidence interval.

**Figure 5:**
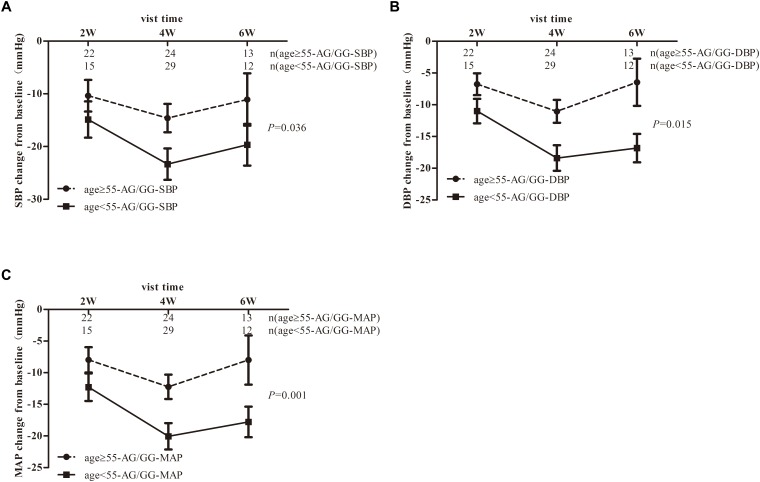
Blood pressure response to α,β-ADR blocker therapy in EH patients stratified by age-specific to TRIB3 genotype. Panels **(A–C)** respectively depicts that SBP-, DBP-, MAP- changes from baseline in EH patients carrying the *TRIB3* (251, A > G) AA and AG/GG genotypes after treatment with α, β-ADRs for 6 weeks. *P*-value were adjusted for baseline BMI, gender, SBP, DBP, total cholesterol, heart rate, triglyceride, HDL, LDL, FBG and other biochemical factors appropriately. Error bar indicated 95% confidence interval. The stratification based on a boundary of age-55 showed the largest difference in blood pressure decrease after analysis of age-50, 55, 60.

As studies showed that α, β-ADRs were all playing a role in the cAMP/cGMP/adrenalin signaling pathways to induce the transcription of eNOS in a time-dependent manner^[18]^, we combined α, β-ADRs treatment groups in the analysis model. However, there was no significant change between AG/GG and AA genotype in the linear mixed model (Figure 3C). Stratified analysis was then used to explore the difference in SBP-, DBP-, MAP-change from baseline after α, β-ADRs therapy. In the sex stratification analysis, BP-change from baseline was significantly reduced for male compared to female in AG/GG genotype (Figure 4). And in the analysis of age stratification, we took 50,55,60 as the boundaries for analysis respectively, and found that 55 was the most appropriate cut-off value. The results showed that people with age younger than 55 years old had more significant antihypertensive effects in AG/GG genotype carriers (Figure 5).

## Discussion

In this study we showed that *TRIB3* (251, A > G) genetic variation had a significant clinical relevance to the efficacy of certain class of antihypertensive drugs in individuals with EH treatment. It has been demonstrated that a better effect on BP response was observed in *TRIB3* Q84R (rs2295490, 251 A > G) AA genotype carriers than AG/GG carriers after taking a routine dosage of ACEI and CCBs antihypertensives. After stratification analysis for age and sex, we found a more significant difference between these two genotype carriers. Studies have shown that CCBs and ACEI have an excellent antioxidant stress effect via increasing eNOS expression and NO synthesis (Kanno et al., 2001; Koyama et al., 2007). *TRIB3*, has been identified affecting insulin action by binding to and inhibiting Akt phosphorylation, which also regulated eNOS and NO. In human and mice, insulin can relax bladder via activating PI3K-AKT-eNOS pathway and lead to a three folds increase in NO synthesis than baseline in AA-but not in *TRIB3* AG/GG genotype carriers (Leiria et al., 2013). Meanwhile, insulin had almost no effect on NO synthesis for AG/GG genotype carriers in human umbilical vein endothelial cells (Andreozzi et al., 2008). Taken together, our results are consistent with the deleterious role of *TRIB3* G allele on NO production, which may lead to a weaker response to ACEI and CCBs antihypertensives.

On the contrary, a better response to ARBs was found on AG/GG genotype carriers than AA genotype carriers. This phenomenon may be interpreted as the different mechanisms of Angiotensin. The published literature has reported that Ang-(1-7) can elevate blood pressure by promoting pro-inflammatory and pro-thrombotic effects. Moreover, Ang IV can activate AT4R, and may contribute to cardiac damage (Ruiz-Ortega et al., 2007; Velkoska et al., 2011). Likewise, Yang et al. (2010) has demonstrated that Ang IV increased blood pressure and reduced renal blood flow through counteracting the presence of AT2 receptors in mice. Other studies revealed that Ang IV can ameliorate AngII-induced cardiac injury via AT4R and protect against acute cerebral ischemia via Ang IV-AT4R, NO-dependent mechanisms (Faure et al., 2006; Yang et al., 2011). As mentioned above, an Ang-(1-7) triggered mechanism involving AT1R-, AT4R-, mass receptors and cGMP-PKG-NO pathway may cause the different antihypertensive effect of ACEI and ARB among individuals induced by *TRIB3* (251, A > G) genetic polymorphism. In addition, ARB could down-regulate *TRIB3* mRNA level and a dual function of ARB on eNOS expression may also lead to the opposite relationship between *TRIB3* G allele and antihypertensive effects of ACEI and ARBs (Zhang et al., 2006; Myojo et al., 2014).

In the ARB and ACEI treatment groups, we noticed that the difference in antihypertensive efficacy between different carriers of *TRIB3* genotypes became more significant as time went on, which may be due to an increase in local eNOS mRNA expression and a decrease in angiotensin II in the LV, leading to the amelioration of myocardial remodeling in hypertensive condition (Kobayashi et al., 2000). Reversed results of ARBs were also shown in the sex stratification. In this drug treatment group, female of AA genotype but not those with G allele have stronger antihypertensive efficacy compared to male. In the treatment of ACEIs, older (age ≥ 55) patients with AA genotype have stronger antihypertensive efficacy compared to younger patients (age < 55). The specific mechanism needs more in-depth discussion and exploration.

In EH patients treated with α, β-ADRs, we did not observe significant difference of antihypertensive effect on response to *TRIB3* genotypes. However, it is interesting to note that *TRIB3* (251, A > G) variance was associated with α, β-ADRs monotherapy in Chinese population with EH in an age-dependent and sex-specific manner. And between the age groups and the sex groups, there is a significant difference in BP-change from baseline only limited to patients with *TRIB3* (251, A > G) AG/GG genotype, while the mechanism is unclear. In addition, our study has shown that among G allele carriers, males or youngers are more likely to have a better antihypertensive effect (Figure 4, 5). This may be attributed to the inconsistent distribution of adrenoceptor in a sex-dependent manner. The regulation of estrogen may also result in greater adrenoceptor blocker reactivity in males than in females (Luksha et al., 2005; Al-Gburi et al., 2017). Researches showed that aging decreases cardiac beta-adrenergic responsiveness in model systems and in humans *in vivo* with multiple mechanisms including downregulation and decreased agonist binding of beta 1-receptors, uncoupling of beta 2-receptors, and abnormal G protein-mediated signal transduction (White et al., 1994).

Reduction in BP by 1–4 mm was also a question be worthy to think about, in the research of Lewington et al. (2002) a 2 mm Hg lower usual SBP was associated with approximately 10% lower stroke mortality and about 7% lower mortality from ischaemic heart disease or other vascular causes in middle age. In general, a 20 mm Hg difference in usual SBP is approximately equivalent in its hazards to a 10 mm Hg difference in usual DBP (Lewington et al., 2002). Therefore, we make hypothesis that for every 1 mm Hg reduction in diastolic blood pressure in the general normotensive population, the mortality rate of vascular-related events can be reduced by 7–10%. For patients with high blood pressure, a decrease of several mmHg of BP may not bring such a big benefit, but we can still assume the benefit exists.

In recent years it became evident that the vast majority of genetic variants in genes of importance for drug treatment are rare, with minor allele frequencies <1%. Also, recent approximations based on analyses of population-scale sequencing projects estimate that such rare variants account for 30–40% of the interindividual variability in drug response (Lauschke and Ingelman-Sundberg, 2018). Some low-frequency genetic variants detected by re-sequencing of *TRIB3* can partially account for cardiovascular clinical outcomes in diabetes. But the association was especially strong among individuals who also carried the common R84 variant (Prudente et al., 2015). Other studies reported that the variation at 5′-untranslated region of the gene may have a strong impact on translational efficiency and serve an important function on gene expression regulating (Hughes, 2006). Several additional infrequent nonsynonymous single-nucleotide polymorphisms have been identified in the genomic region encompassing the human *TRIB3* gene (dbSNP^2^) whose function and potential biological relevance have never, so far, been addressed. These may lead us to speculate that rare and common variants in *TRIB3* may act together to alter drug efficacy.

Our study was a well-controlled drug clinical trial in which patients received monotherapy. Nevertheless, some limitations should be considered in the interpretation of our findings. On the one hand, diet habits, lifestyle factors may affect the antihypertensive effect of drugs. Such as a daily mean of 30 min of vigorous exercise, a DASH style diet, modest alcohol intake up to 10 g/day, non-narcotic analgesic use less than once per week, and intake of 400 μg/day or more of supplemental folic acid may lead to lower risk of hypertension (Forman et al., 2009). These factors also have the potential to make patients have a distinguishing antihypertensive effect. However, our results do not exclude the influence of these factors because of the lack of these information. On the other hand, polymorphisms of other genes may also be involved in the interaction between heredity and drug efficacy or affect vascular function (He et al., 2015; Shen et al., 2019).

## Conclusion

In conclusion, the results of the present study suggest that the *TRIB3* (251, A > G) polymorphism was associated with the response of antihypertensive drugs therapy in Chinese EH patients,. Although this data may prove *TRIB3* (251, A > G) genetic variation is useful in clinical therapy, combining other genetic markers to identify individuals at high risk of cardiovascular abnormalities may bring a better clinical outcome. Further pharmacogenomics studies on *TRIB3* merit attention.

## Author Contributions

JZ, FH, JL, LX, and WZ were responsible for the study design. JZ, FH, and BS were responsible for the experiments. JZ and FH did the data processing and statistical analysis. JZ, FH, and BS wrote and interpreted the manuscript. FH and BS were responsible for data management and contributed reagents and materials. RL, YG, HR, YS, XC, ZL, HZ, SD, and HX calibrated and discussed the manuscripts.

## Conflict of Interest Statement

The authors declare that the research was conducted in the absence of any commercial or financial relationships that could be construed as a potential conflict of interest.

## Footnotes

1 http://www.chictr.org.cn

2 www.ncbi.nlm.nih.gov/snp

## Supplementary Material

FIGURE S1 Blood pressure response to CCBs, ARBs, and ACEI therapy in EH patients stratified by age-specific to TRIB3 genotype. **A–C** respectively depicts that BP changes from baseline in EH patients carrying the *TRIB3* (251, A>G) AA and AG/GG genotypes after treatment with CCBs, ARBs, and ACEI for 6 weeks respectively. *P*-value were adjusted for baseline BMI, sex, SBP, DBP, total cholesterol, heart rate, triglyceride, HDL, LDL, FBG, and other biochemical factors appropriately. Error bar indicated 95% confidence interval. The stratification based on a boundary of age-55 showed the largest difference in blood pressure decrease after analysis of age-50, 55, 60.

FIGURE S2 Blood pressure response to CCBs, ARBs, and ACEI therapy in EH patients stratified by sex dependent of TRIB3 genotype. **A–C** respectively depicts that BP changes from baseline in EH patients carrying the *TRIB3* (251, A>G) AA and AG/GG genotypes after treatment with CCBs, ARBs, and ACEI for 6 weeks respectively. *P*-value were adjusted for baseline age, BMI, SBP, DBP, total cholesterol, heart rate, triglyceride, HDL, LDL, FBG, and other biochemical factors appropriately. Error bar indicated 95% confidence interval.

## References

[B1] Al-Gburi S. Deussen A. Zatschler B. Weber S. Künzel S. El-Armouche A. (2017). Sex-difference in expression and function of beta-adrenoceptors in macrovessels: role of the endothelium. *Basic Res. Cardiol.* 112:29. 10.1007/s00395-017-0617-2 28389717

[B2] Andreozzi F. Formoso G. Prudente S. Hribal M. L. Pandolfi A. Bellacchio E. (2008). TRIB3 R84 variant is associated with impaired insulin-mediated nitric oxide production in human endothelial cells. *Arterioscler. Thromb. Vasc. Biol.* 28 1355–1360. 10.1161/atvbaha.108.162883 18436806

[B3] Chan M. C. Nguyen P. H. Davis B. N. Ohoka N. Hayashi H. Du K. (2007). A novel regulatory mechanism of the bone morphogenetic protein (BMP) signaling pathway involving the carboxyl-terminal tail domain of BMP type II receptor. *Mol. Cell Biol.* 27 5776–5789. 10.1128/mcb.00218-07 17576816 PMC1952124

[B4] Chan M. C. Weisman A. S. Kang H. Nguyen P. H. Hickman T. Mecker S. V. (2011). The amiloride derivative phenamil attenuates pulmonary vascular remodeling by activating NFAT and the bone morphogenetic protein signaling pathway. *Mol. Cell Biol.* 31 517–530. 10.1128/mcb.00884-10 21135135 PMC3028626

[B5] Chen R. Iwai M. Wu L. Suzuki J. Min L. J. Shiuchi T. (2003). Important role of nitric oxide in the effect of angiotensin-converting enzyme inhibitor imidapril on vascular injury. *Hypertension* 42 542–547. 10.1161/01.hyp.0000092440.52239.39 12963679

[B6] Chen Y. L. Pan A. W. Hsiung P. C. Chung L. Lai J. S. Shur-Fen Gau S (2015). Life adaptation skills training (LAST) for persons with depression: a randomized controlled study. *J. Affect. Disord.* 185 108–114. 10.1016/j.jad.2015.06.022 26162281

[B7] Cui A. D. Gai N. N. Zhang X. H. Jia K. Z. Yang Y. L. Song Z. J. (2012). Decreased serum obestatin consequent upon TRIB3 Q84R polymorphism exacerbates carotid atherosclerosis in subjects with metabolic syndrome. *Diabetol. Metab. Syndr.* 4:52. 10.1186/1758-5996-4-52 23245314 PMC3573955

[B8] Du K. Herzig S. Kulkarni R. N. Montminy M. (2003). TRB3: a tribbles homolog that inhibits Akt/PKB activation by insulin in liver. *Science* 300 1574–1577. 10.1126/science.1079817 12791994

[B9] Faure S. Chapot R. Tallet D. Javellaud J. Achard J. M. Oudart N. (2006). Cerebroprotective effect of angiotensin IV in experimental ischemic stroke in the rat mediated by AT(4) receptors. *J. Physiol. Pharmacol.* 57 329–342. 17033088

[B10] Fischer Z. Das R. Shipman A. Fan J. Y. Pence L. Bouyain S. (2017). A Drosophila model of insulin resistance associated with the human TRIB3 Q/R polymorphism. *Dis. Model. Mech.* 10 1453–1464. 10.1242/dmm.030619 29025897 PMC5769606

[B11] Forman J. P. Stampfer M. J. Curhan G. C. (2009). Diet and lifestyle risk factors associated with incident hypertension in women. *JAMA* 302 401–411. 10.1001/jama.2009.1060 19622819 PMC2803081

[B12] Formoso G. Di Tomo P Andreozzi F. Succurro E. Di Silvestre S. Prudente S. (2011). The TRIB3 R84 variant is associated with increased carotid intima-media thickness in vivo and with enhanced MAPK signalling in human endothelial cells. *Cardiovasc. Res.* 89 184–192. 10.1093/cvr/cvq255 20693163

[B13] Gong H. P. Wang Z. H. Jiang H. Fang N. N. Li J. S. Shang Y. Y. (2009). TRIB3 functional Q84R polymorphism is a risk factor for metabolic syndrome and carotid atherosclerosis. *Diabetes Care* 32 1311–1313. 10.2337/dc09-0061 19389818 PMC2699701

[B14] He F. Liu M. Chen Z. Liu G. Wang Z. Liu R. (2016). Assessment of human tribbles homolog 3 genetic variation (rs2295490) effects on type 2 diabetes patients with glucose control and blood pressure lowering treatment. *EBioMedicine* 13 181–189. 10.1016/j.ebiom.2016.10.025 27793583 PMC5264271

[B15] He F. Luo J. Zhang Z. Luo Z. Fan L. He Y. (2015). The RGS2 (-391, C > G) genetic variation correlates to antihypertensive drug responses in Chinese patients with essential hypertension. *PLoS One* 10:e0121483. 10.1371/journal.pone.0121483 25849301 PMC4388730

[B16] He F. Shu Y. Wang X. Liu X. Liu G. Chen Z. (2018). Intensive glucose control reduces the risk effect of TRIB3, SMARCD3, and ATF6 genetic variation on diabetic vascular complications. *Front. Pharmacol.* 9:1422. 10.3389/fphar.2018.01422 30618737 PMC6297143

[B17] Hughes T. A. (2006). Regulation of gene expression by alternative untranslated regions. *Trends Genet* 22 119–122. 10.1016/j.tig.2006.01.001 16430990

[B18] Kanno S. Wu Y. J. Lee P. C. Billiar T. R. Ho C. (2001). Angiotensin-converting enzyme inhibitor preserves p21 and endothelial nitric oxide synthase expression in monocrotaline-induced pulmonary arterial hypertension in rats. *Circulation* 104 945–950. 10.1161/hc3401.093155 11514384

[B19] Kim S. Hata A. Kang H. (2014). Down-regulation of miR-96 by bone morphogenetic protein signaling is critical for vascular smooth muscle cell phenotype modulation. *J. Cell Biochem.* 115 889–895. 10.1002/jcb.24730 24375867 PMC5215840

[B20] Kimura Y. Hirooka Y. Sagara Y. Sunagawa K. (2007). Long-acting calcium channel blocker, azelnidipine, increases endothelial nitric oxide synthase in the brain and inhibits sympathetic nerve activity. *Clin. Exp. Hypertens.* 29 13–21. 10.1080/10641960601096745 17190727

[B21] Kiss-Toth E. Bagstaff S. M. Sung H. Y. Jozsa V. Dempsey C. Caunt J. C. (2004). Human tribbles, a protein family controlling mitogen-activated protein kinase cascades. *J. Biol. Chem.* 279 42703–42708. 10.1074/jbc.M407732200 15299019

[B22] Kobayashi N. Hara K. Watanabe S. Higashi T. Matsuoka H. (2000). Effect of imidapril on myocardial remodeling in L-NAME–induced hypertensive rats is associated with gene expression of NOS and ACE mRNA. *Am. J. Hypertens.* 13 199–207. 10.1016/S0895-7061(99)00117-X 10701821

[B23] Kobayashi N. Mita S. Yoshida K. Honda T. Kobayashi T. Hara K. (2003). Celiprolol activates eNOS through the PI3K-Akt pathway and inhibits VCAM-1 Via NF-κB induced by oxidative stress. *Hypertension* 42 1004–1013. 10.1161/01.hyp.0000097547.35570.70 14557279

[B24] Koyama Y. Takeishi Y. Takahashi H. Shishido T. Arimoto T. Niizeki T. (2007). Azelnidipine inhibits H2O2-induced cell death in neonatal rat cardiomyocytes. *Cardiovasc. Drugs Ther.* 21 69–72. 10.1007/s10557-007-6008-4 17318380

[B25] Lauschke V. M. Ingelman-Sundberg M. (2018). How to consider rare genetic variants in personalized drug therapy. *Clin. Pharmacol. Ther.* 103 745–748. 10.1002/cpt.976 29313952

[B26] Leiria L. O. Sollon C. Báu F. R. Mónica F. Z. D’Ancona C. L. De Nucci G. (2013). Insulin relaxes bladder via PI3K/AKT/eNOS pathway activation in mucosa: unfolded protein response-dependent insulin resistance as a cause of obesity-associated overactive bladder. *J. Physiol.* 591 2259–2273. 10.1113/jphysiol.2013.251843 23478138 PMC3650693

[B27] Lewington S. Clarke R. Qizilbash N. Peto R. Collins R. (2002). Age-specific relevance of usual blood pressure to vascular mortality: a meta-analysis of individual data for one million adults in 61 prospective studies. *Lancet* 360 1903–1913. 10.1016/S0140-6736(02)11911-8 12493255

[B28] Luksha L. Poston L. Gustafsson J. A. Aghajanova L. Kublickiene K. (2005). Gender-specific alteration of adrenergic responses in small femoral arteries from estrogen receptor-beta knockout mice. *Hypertension* 46 1163–1168. 10.1161/01.HYP.0000185648.48498.c1 16216990

[B29] Messerli F. H. Williams B. Ritz E. (2007). Essential hypertension. *Lancet* 370 591–603. 10.1016/s0140-6736(07)61299-9 17707755

[B30] Myojo M. Nagata D. Fujita D. Kiyosue A. Takahashi M. Satonaka H. (2014). Telmisartan activates endothelial nitric oxide synthase via Ser1177 phosphorylation in vascular endothelial cells. *PLoS One* 9:e96948. 10.1371/journal.pone.0096948 24827148 PMC4020804

[B31] Patel A. Advance Collaborative Group MacMahon S. Chalmers J. Neal B. Woodward M., (2007). Effects of a fixed combination of perindopril and indapamide on macrovascular and microvascular outcomes in patients with type 2 diabetes mellitus (the ADVANCE trial): a randomised controlled trial. *Lancet* 370 829–840. 10.1016/s0140-6736(07)61303-8 17765963

[B32] Prudente S. Bailetti D. Mendonca C. Mannino G. C. Fontana A. Andreozzi F. (2015). Infrequent TRIB3 coding variants and coronary artery disease in type 2 diabetes. *Atherosclerosis* 242 334–339. 10.1016/j.atherosclerosis.2015.07.030 26253791 PMC4872508

[B33] Prudente S. Hribal M. L. Flex E. Turchi F. Morini E. De Cosmo S. (2005). The functional Q84R polymorphism of mammalian Tribbles homolog TRB3 is associated with insulin resistance and related cardiovascular risk in Caucasians from Italy. *Diabetes* 54 2807–2811. 10.2337/diabetes.54.9.2807 16123373

[B34] Prudente S. Trischitta V. (2015). The TRIB3 Q84R polymorphism, insulin resistance and related metabolic alterations. *Biochem. Soc. Trans.* 43 1108–1111. 10.1042/bst20150115 26517932

[B35] Qin R. R. Song M. Li Y. H. Wang F. Zhou H. M. Liu M. H. (2017). Association of increased serum sema3E with TRIB3 Q84R polymorphism and carotid atherosclerosis in metabolic syndrome. *Ann. Clin. Lab. Sci.* 47 47–51. 28249916

[B36] Ruiz-Ortega M. Esteban V. Egido J. (2007). The regulation of the inflammatory response through nuclear factor-kappab pathway by angiotensin IV extends the role of the renin angiotensin system in cardiovascular diseases. *Trends Cardiovasc. Med.* 17 19–25. 10.1016/j.tcm.2006.10.003 17210474

[B37] Shen J. Liu M. Xu J. Sun B. Xu H. Zhang W. (2019). ARL15 overexpression attenuates high glucose-induced impairment of insulin signaling and oxidative stress in human umbilical vein endothelial cells. *Life Sci.* 220 127–135. 10.1016/j.lfs.2019.01.030 30682341

[B38] Stojkov N. J. Baburski A. Z. Bjelic M. M. Sokanovic S. J. Mihajlovic A. I. Drljaca D. M. (2014). In vivo blockade of alpha1-adrenergic receptors mitigates stress-disturbed cAMP and cGMP signaling in Leydig cells. *Mol. Hum. Reprod.* 20 77–88. 10.1093/molehr/gat052 23894150

[B39] Stojkov N. J. Janjic M. M. Kostic T. S. Andric S. A. (2013). Orally applied doxazosin disturbed testosterone homeostasis and changed the transcriptional profile of steroidogenic machinery, cAMP/cGMP signalling and adrenergic receptors in Leydig cells of adult rats. *Andrology* 1 332–347. 10.1111/j.2047-2927.2012.00035.x 23413145

[B40] Sun B. He F. Sun L. Zhou J. Shen J. Xu J. (2019). Cause-specific risk of major adverse cardiovascular outcomes and hypoglycemic in patients with type 2 diabetes: a multicenter prospective cohort study. *Endocrine* 63 44–51. 10.1007/s12020-018-1715-0 30121774

[B41] Velkoska E. Dean R. G. Griggs K. Burchill L. Burrell L. M. (2011). Angiotensin-(1-7) infusion is associated with increased blood pressure and adverse cardiac remodelling in rats with subtotal nephrectomy. *Clin. Sci.* 120 335–345. 10.1042/cs20100280 21091432 PMC3018845

[B42] Wain L. V. Verwoert G. C. O’Reilly P. F. Shi G. Johnson T. Johnson A. D. (2011). Genome-wide association study identifies six new loci influencing pulse pressure and mean arterial pressure. *Nat. Genet.* 43 1005–1011. 10.1038/ng.922 21909110 PMC3445021

[B43] West J. Fagan K. Steudel W. Fouty B. Lane K. Harral J. (2004). Pulmonary hypertension in transgenic mice expressing a dominant-negative BMPRII gene in smooth muscle. *Circ. Res.* 94 1109–1114. 10.1161/01.res.0000126047.82846.20 15031260

[B44] White M. Roden R. Minobe W. Khan M. F. Larrabee P. Wollmering M. (1994). Age-related changes in beta-adrenergic neuroeffector systems in the human heart. *Circulation* 90 1225–1238. 10.1161/01.CIR.90.3.1225 8087932

[B45] Yang H. Zeng X. J. Wang H. X. Zhang L. K. Dong X. L. Guo S. (2011). Angiotensin IV protects against angiotensin II-induced cardiac injury via AT4 receptor. *Peptides* 32 2108–2115. 10.1016/j.peptides.2011.09.015 21963909

[B46] Yang R. Walther T. Gembardt F. Smolders I. Vanderheyden P. Albiston A. L. (2010). Renal vasoconstrictor and pressor responses to angiotensin IV in mice are AT1a-receptor mediated. *J. Hypertens.* 28 487–494. 10.1097/HJH.0b013e3283343250 19907343

[B47] Yu Q. Gao F. Ma X. L. (2011). Insulin says NO to cardiovascular disease. *Cardiovasc. Res.* 89 516–524. 10.1093/cvr/cvq349 21051417

[B48] Zhang W. Yang Z. Li X. Wen J. Zhang H. Wang S. (2015). The functional Q84R polymorphism of TRIB3 gene is associated with diabetic nephropathy in Chinese type 2 diabetic patients. *Gene* 555 357–361. 10.1016/j.gene.2014.11.031 25447894

[B49] Zhang W. Zhong M. Tang M. X. Ma X. Miao Y. Sun H. (2006). Effect of valsartan on Tribble 3 gene expression in rats with experimental diabetic cardiomyopathy. *Zhonghua Xin Xue Guan Bing Za Zhi* 34 212–216. 16630451

[B50] Zou T. Liu W. J. Li S. D. Zhou W. Yang J. F. Zou C. G. (2011). TRB3 mediates homocysteine-induced inhibition of endothelial cell proliferation. *J. Cell Physiol.* 226 2782–2789. 10.1002/jcp.22554 21935927

