# Supplementary figures and images for: Polytropic Influence of TRIB3 rs2295490 Genetic Polymorphism on Response to Antihypertensive Agents in Patients With Essential Hypertension
Source: Front Pharmacol. 2019 Mar 27;10:236. doi: 10.3389/fphar.2019.00236 (PMC6445854; doi:10.3389/fphar.2019.00236)

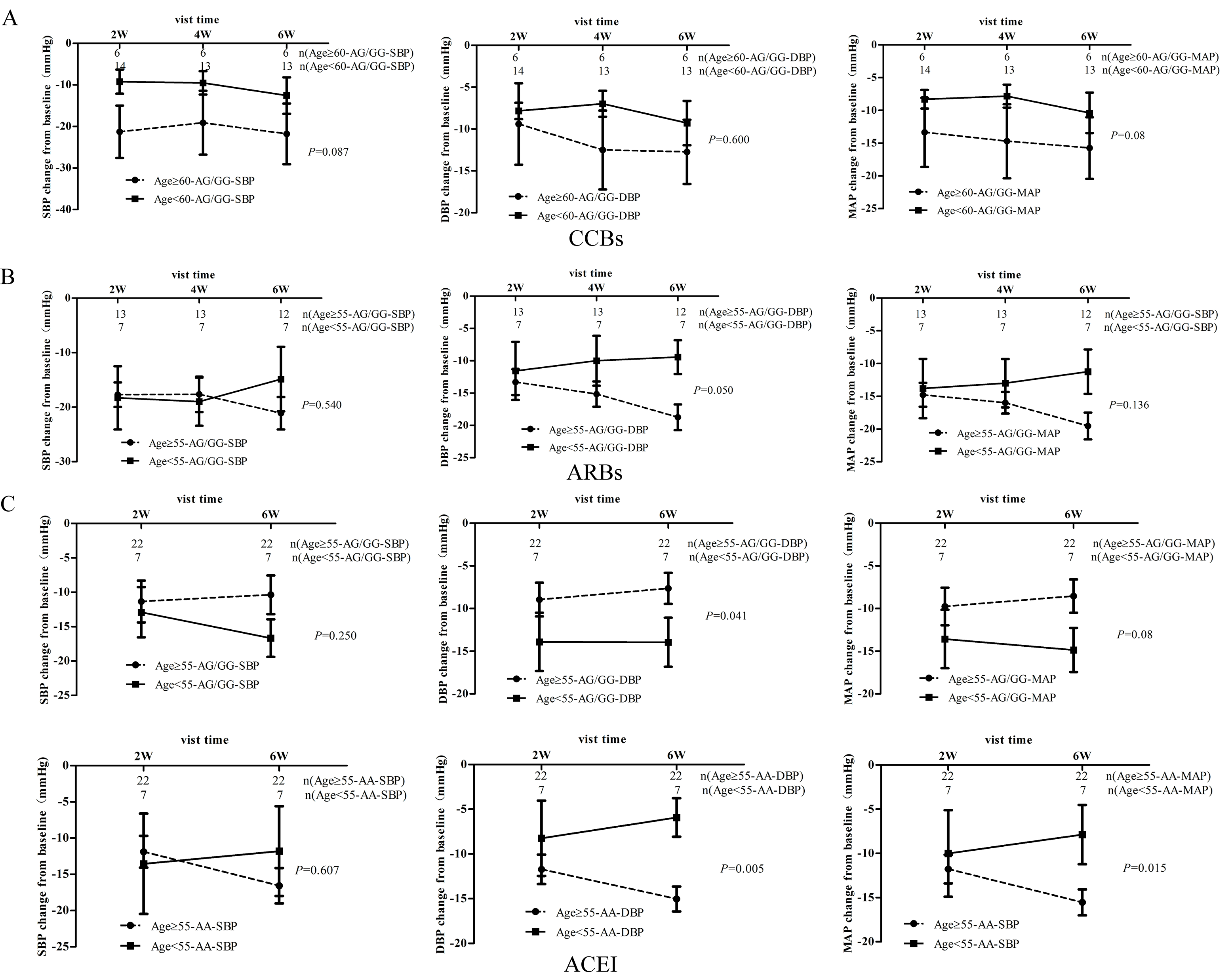

Supplement: FIGURE S1 — Blood pressure response to CCBs, ARBs, and ACEI therapy in EH patients stratified by age-specific to TRIB3 genotype. A–C respectively depicts that BP changes from baseline in EH patients carrying the TRIB3 (251, A>G) AA and AG/GG genotypes after treatment with CCBs, ARBs, and ACEI for 6 weeks respectively. P-value were adjusted for baseline BMI, sex, SBP, DBP, total cholesterol, heart rate, triglyceride, HDL, LDL, FBG, and other biochemical factors appropriately. Error bar indicated 95% confidence interval. The stratification based on a boundary of age-55 showed the largest difference in blood pressure decrease after analysis of age-50, 55, 60. [file Image_1.TIF]
